# Supplementary material for: Seeds harvested during mowing from semi-natural grasslands as an ad hoc but effective solution for grassland restoration
Source: PeerJ. 2022 Jul 13;10:e13621. doi: 10.7717/peerj.13621 (PMC9288168; doi:10.7717/peerj.13621)
Supplement: Table S2 [file peerj-10-13621-s001.pdf]

Table S2.1 Frequencies of each species in each trial and species coverage in field experiments

Species	Seed check mower	Pot experiment mower			Field 1 experiment mower		Field 2 experiment mower	
	Frequency (10 samples)	Sowed (July 2020)		Sowed (October 2020)	Frequency	Cover	Frequency	Cover
		Frequency (October 2020)	Frequency (May 2021)	Frequency (May 2021)				
Agrostis capillaris L.	-	1	0.9	1	-	-	-	-
Alopecurus pratensis L.	1	0.3	0.1	0.2	-	-	-	-
Anthemis tinctoria L.	-	-	-	-	0.5	1.5	-	-
Anthoxanthum odoratum L.	0.9	0.4	-	-	-	-	-	-
Anthriscus sylvestris (L.) Hoffm.	0.1	-	-	-	-	-	-	-
Arabidopsis thaliana (L.) Heynh.	-	-	-	-	0.75	3.7	-	-
Arrhenatherum elatius (L.) J.Presl & C.Presl	0.9	0.7	0.9	1	1	41.3	0.5	20.0
Artemisia vulgaris L.	-	0.4	0.2	0.2	0.5	1	-	-
Bromus hordeaceus L.	0.3	-	-	-	0.5	3	0.5	2.5
Bromus sterilis L.	-	-	-	-	1	3.8	-	-
Campanula patula L.	-	-	-	0.3	-	-	-	-
Capsella bursa-pastoris (L.) Medik.	-	-	-	-	0.5	3.5	0.5	3.0
Cerastium fontanum Baumg.	-	0.1	0.5	0.7	-	-	-	-
Cirsium arvense (L.) Scop.	-	-	-	0.2	1	2		
Dactylis glomerata L.	1	1	1	1	0.25	2	0.5	1.5
Deschampsia caespitosa (L.) P. Beauv.	-	0.2	-	-	-	-	-	-
Descurainia sophia (L.) Webb ex Prantl	-	-	-	-	0.25	1	-	-
Elymus repens (L.) Gould	-	0.9	0.8	0.7	0.5	1	0.25	2.0
Festuca pratensis Huds.	1	0.2	-	0.1	-	-	-	-
Festuca rubra L.	1	0.7	0.5	0.7	-	-	-	-
Galium mollugo L.	0.6	-	-	-	-	-	0.25	2.0
Geranium pusillum L.	-	-	-	-	1	13.8	0.25	2.0
Holcus lanatus L.	0.9	0.9	1	1	1	1.3	0.5	1.0
Leucanthemum vulgare Lam.	0.2	0.5	0.5	0.1	0.25	2	0.25	1.0
Lolium perenne L.	0.3	1	0.5	0.3	-	-	1	32.5
Papaver orientale L.	-	-	-	-	0.25	2	-	-
Papaver rhoeas L.	-	-	-	-	1	2	-	-
Phacelia tanacetifolia Benth.	-	-	-	-	0.25	1	-	-
Phleum pratense L.	0.6	0.1	-	-	0.25	2	0.25	2.0
Plantago lanceolata L.	-	0.1	-	-	0.25	2	0.5	1.0
Poa pratensis L.	0.9	0.9	1	1	1	1.8	0.5	4.0
Polygonum persicaria L.	-	0.1	0.2	0.2	-	-	-	-
Ranunculus acris L.	-	-	0.1	-	-	-	-	-
Ranunculus repens L.	0.1	-	-	0.2	-	-	-	-
Rhinanthus minor L.	0.1	-	0.1	-	-	-	0.5	2.5
Rumex acetosa L.	0.9	0.4	0.6	0.7	-	-	0.25	4.0
Rumex obtusifolius L.	0.1	0.1	-	-	-	-	0.25	1.0
Sisymbrium officinale (L.) Scop.	-	-	-	-	0.25	3	-	-
Solidago gigantea Aiton	-	-	-	-	0.75	3.3	-	-
Sonchus asper (L.) Hill	-	-	-	-	1	2.5	-	-
Stellaria holostea L.	-	0.1	-	-	-	-	-	-
Stellaria media (L.) Cirillo	-	0.3	0.5	0.4	-	-	0.25	2.0
Taraxacum officinale (L.) F.H.Wigg	-	0.1	0.1	0.1	1	1.8	1	5.8
Trifolium dubium Sibth.	-	-	-	-	0.5	3	-	-
Trifolium pratense L.	-	-	-	-	0.25	2	0.5	2.0
Trifolium repens L.	-	-	-	0.1	-	-	1	9.5
Tripleurospermum maritimum (L.) W.D.J. Koch	-	-	-	-	0.25	2	-	-
Trisetum flavescens (L.) P.Beauv.	0.8	0.1	-	-	1	4.3	-	-
Unrecognised species from Poaceace	-	-	0.1	-	-	-	-	-
Urtica dioica L.	-	0.2	0.1	0.1	-	-	-	-

Veronica chamaedrys L.	-	-	-	-	1	1.3	0.5	2.0
Veronica verna L.	-	-	-	-	1	4.3	0.75	2.3
Vicia hirsuta (L.) Gray	-	-	-	-	0.5	3	0.25	3.0
Viola arvensis Murray	-	-	-	-	0.5	3	-	-
Arenaria serpyllifolia L.	-	-	-	-	-	-	0.5	2.5
Cerastium vulgare Hartm.	-	-	-	-	-	-	1	10.3
Consolida regalis Gray	-	-	-	-	-	-	0.25	0.5
Convolvulus arvensis L.	-	-	-	-	-	-	0.25	1.0
Dianthus deltoides L.	-	-	-	-	-	-	0.75	1.0
Erigeron annuus (L.) Desf.	-	-	-	-	-	-	0.25	5.0
Hypochaeris radicata L.	-	-	-	-	-	-	0.25	2.0
Medicago lupulina L.	-	-	-	-	-	-	0.75	3.0
Papaver dubium L.	-	-	-	-	-	-	0.5	1.5
Polygonum hydropiper L. (*Persicaria hydropiper (L.) Del*	-	-	-	-	-	-	0.75	3.3
Tanacetum vulgare L.	-	-	-	-	-	-	0.25	1.0
Trifolium arvense L.	-	-	-	-	-	-	1	3.0
Trifolium incarnatum L.	-	-	-	-	-	-	0.5	1.5
Vicia sativa L.	-	-	-	-	-	-	0.75	2.7

Table S2.2 Number of seedlings in petridish germination test - Seeds sown 15th October, 2

Date	22th October 2020			29th October 2020			5th November 2020			12th November 2020					
Replicate	Grass	Other	Sub total1	Grass	Other	Sub total2	Grass	Other	Sub total3	Grass	Other	Sub total4	Gross Total	Total number of seed per 1g	Germination Percentage (%)
R1	322	6	328	112	0	112	93	0	93	22	0	22	555	1267.1	43.80
R2	315	2	317	90	1	91	71	0	71	22	0	22	501	1267.1	39.54
R3	360	1	361	96	1	97	45	0	45	23	0	23	526	1267.1	41.51
R4	326	3	329	89	0	89	77	0	77	22	0	22	517	1267.1	40.80
R5	304	2	306	136	1	137	86	0	86	35	0	35	564	1267.1	44.51
R6	306	1	307	115	0	115	73	0	73	33	0	33	528	1267.1	41.67
R7	308	2	310	118	0	118	64	0	64	27	0	27	519	1267.1	40.96
R8	325	3	328	115	1	116	82	0	82	30	0	30	556	1267.1	43.88
R9	329	2	331	103	0	103	65	0	65	22	0	22	521	1267.1	41.12
R10	332	2	334	95	0	95	72	0	72	18	0	18	519	1267.1	40.96
Total G/O	3227	24	3251	1069	4	1073	728	0	728	254	0	254	5306	1267.1	418.75
									Total				5306		
									Average				530.6	1267.1	41.88

19th November 2020			26th November 2020					
Grass	Other	Sub total5	Grass	Other	Sub total5	Gross Total	Total number of seed per 1g	Germination Percentage (%)
4	0	4	5	0	5	564	1267.1	44.51
11	0	11	2	0	2	514	1267.1	40.57
3	0	3	1	0	1	530	1267.1	41.83
6	0	6	4	0	0	523	1267.1	41.28
7	0	7	8	0	4	575	1267.1	45.38
2	0	2	0	0	8	538	1267.1	42.46
5	0	5	1	0	1	525	1267.1	41.43
4	0	4	4	0	4	564	1267.1	44.51
8	0	8	0	0	0	529	1267.1	41.75
10	0	10	4	0	4	533	1267.1	42.06
60	0	60	29	0	29	5395	12671	425.78
					Avarage	539.5	1267.1	42.58

Table S2.3 Number of seedlings in petridish germination test - Seeds sown 15th March, 20

Date	22th March 2021			29th March 2021			6th April 2021			12th April 2021					
Replicate	Grass	Other	Sub total1	Grass	Other	Sub total2	Grass	Other	Sub total3	Grass	Other	Sub total4	Gross Total	Total number of seed per 1g	Germination Percentage (%)
R1	328	0	328	89	0	89	87	0	87	14	0	14	518	1267.1	40.88
R2	358	1	359	72	1	73	70	0	70	10	0	10	512	1267.1	40.41
R3	321	2	323	83	0	83	88	0	88	11	0	11	505	1267.1	39.85
R4	403	1	404	65	0	65	79	0	79	8	0	8	556	1267.1	43.88
R5	354	0	354	70	0	70	57	0	57	10	0	10	491	1267.1	38.75
R6	404	0	404	107	0	107	63	0	63	21	0	21	595	1267.1	46.96
R7	379	2	381	111	0	111	76	0	76	11	1	12	580	1267.1	45.77
R8	349	0	349	127	1	128	103	0	103	10	0	10	590	1267.1	46.56
R9	344	1	345	121	1	122	87	0	87	7	0	7	561	1267.1	44.27
R10	306	1	307	165	1	166	95	1	96	12	0	12	581	1267.1	45.85
Total G/O	3546	8	3554	1010	4	1014	805	1	806	114	1	115	5489	1267.1	433.19
									Total				5489		
									Average				548.9		43.32

19th April 2021			26th April 2021					
Grass	Other	Sub total5	Grass	Other	Sub total5	Gross Total	Total number of seed per 1g	Germination Percentage (%)
5	0	5	3	0	3	526	1267.1	41.51
5	0	5	4	0	4	521	1267.1	41.12
5	0	5	2	0	2	512	1267.1	40.41
7	0	7	4	0	4	567	1267.1	44.75
4	0	4	2	0	2	497	1267.1	39.22
6	0	6	4	0	4	605	1267.1	47.75
7	0	7	5	0	5	592	1267.1	46.72
10	0	10	7	0	7	607	1267.1	47.90
2	0	2	2	0	2	565	1267.1	44.59
7	0	7	1	0	1	589	1267.1	46.48
58	0	58	34	0	34	5581	1267.1	440.45
					Avarage	558.1	1267.1	44.05

Table S2.4 Number of seedlings in petridish germination test - Seeds sown in 28th July, 20

Date	3rd August 2020			11th August 2020			18th August 2020			24th August 2020					
Replicate	Grass	Other	Sub total1	Grass	Other	Sub total2	Grass	Other	Sub total3	Grass	Other	Sub total4	Gross Total	Total number of seed per 1g	Germination Percentage (%)
R1	76	0	76	252	0	252	76	0	76	35	0	35	439	1382	31.77
R2	70	0	70	210	1	211	77	0	77	50	0	50	408	1386	29.44
R3	75	1	76	213	1	214	92	0	92	34	0	34	416	1526	27.26
R4	79	0	79	201	1	202	98	0	98	58	1	59	438	1248	35.10
R5	86	0	86	201	2	203	54	0	54	51	0	51	394	962	40.96
R6	98	1	99	248	2	250	85	0	85	57	0	57	491	1292	38.00
R7	113	1	114	239	3	242	58	0	58	53	0	53	467	1205	38.76
R8	121	0	121	264	1	265	83	0	83	48	0	48	517	1290	40.08
R9	113	0	113	261	1	262	72	0	72	42	0	42	489	1215	40.25
R10	104	1	105	229	1	230	72	0	72	46	0	46	453	1165	38.88
Total G/O	935	4	939	2318	13	2331	767	0	767	474	1	475	4512	12671	360.48
									Total				4512		
									Average				451.2	1267.1	36.05

1st September 2020			8th September 2020					
Grass	Other	Sub total5	Grass	Other	Sub total5	Gross Total	Total number of seed per 1g	Germination Percentage (%)
25	0	25	6	0	6	470	1382	34.01
38	0	38	8	0	8	454	1386	32.76
68	0	68	18	0	18	502	1526	32.90
66	0	66	16	0	16	520	1248	41.67
49	0	49	9	0	9	452	962	46.99
56	1	57	13	0	13	561	1292	43.42
48	0	48	6	0	6	521	1205	43.24
39	0	39	7	0	7	563	1290	43.64
30	0	30	10	0	10	529	1215	43.54
26	0	26	9	0	9	488	1165	41.89
445	1	446	102	0	102	5060	12671	404.04
					Avarage	506	1267.1	40.40

Table S2.5 Number of seeds, seeds weight and residual in 1-g of seed sample

Sample No	R1	R2	R3	R4	R5	R6	R7	R8	R9	R10	R11	R12	R13	R14	R15	R16	R17	R18	R19	R20
Number of filled seeds (1g):	1382	1386	1526	1248	962	1292	1205	1290	1215	1165										
Total seeds weight (g):	0.8695	0.9097	0.9378	0.9203	0.77	0.9254	0.9068	0.9062	0.8932	0.9355	0.903	0.9362	0.8732	0.9073	0.9137	0.9375	0.89346	0.918	0.8478	0.923
Amount of debries (g):	0.1305	0.0903	0.0622	0.0797	0.23	0.0746	0.0932	0.0938	0.1068	0.0645	0.097	0.0638	0.1268	0.0927	0.0863	0.0625	0.10654	0.082	0.1522	0.077
