# Supplementary figures and images for: Seeds harvested during mowing from semi-natural grasslands as an ad hoc but effective solution for grassland restoration
Source: PeerJ. 2022 Jul 13;10:e13621. doi: 10.7717/peerj.13621 (PMC9288168; doi:10.7717/peerj.13621)

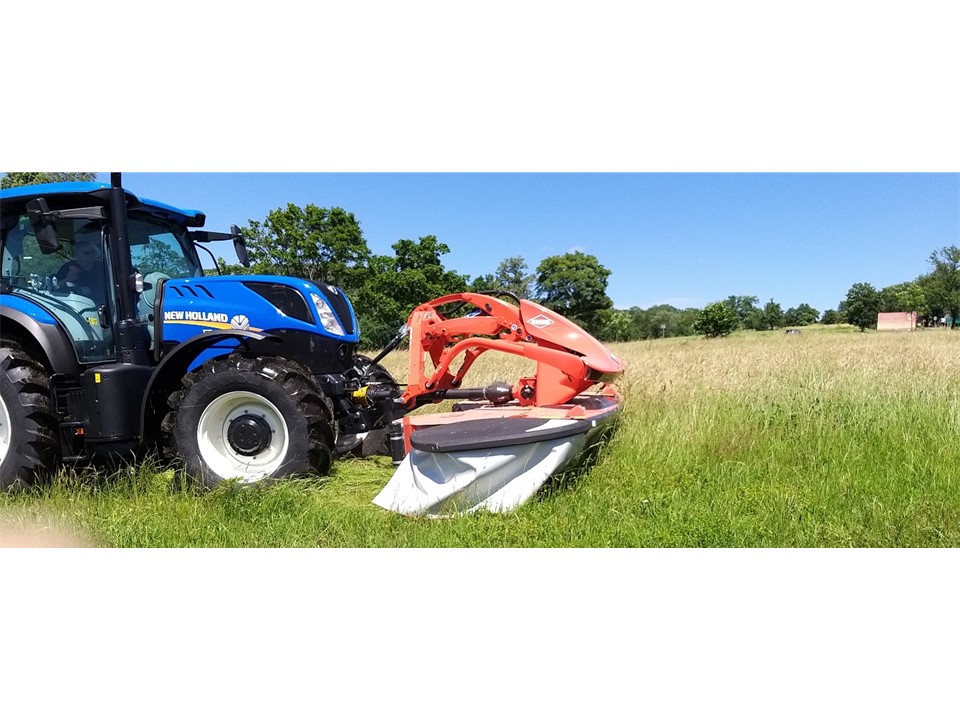

Supplement: Figure S1 [file peerj-10-13621-s002.jpg]

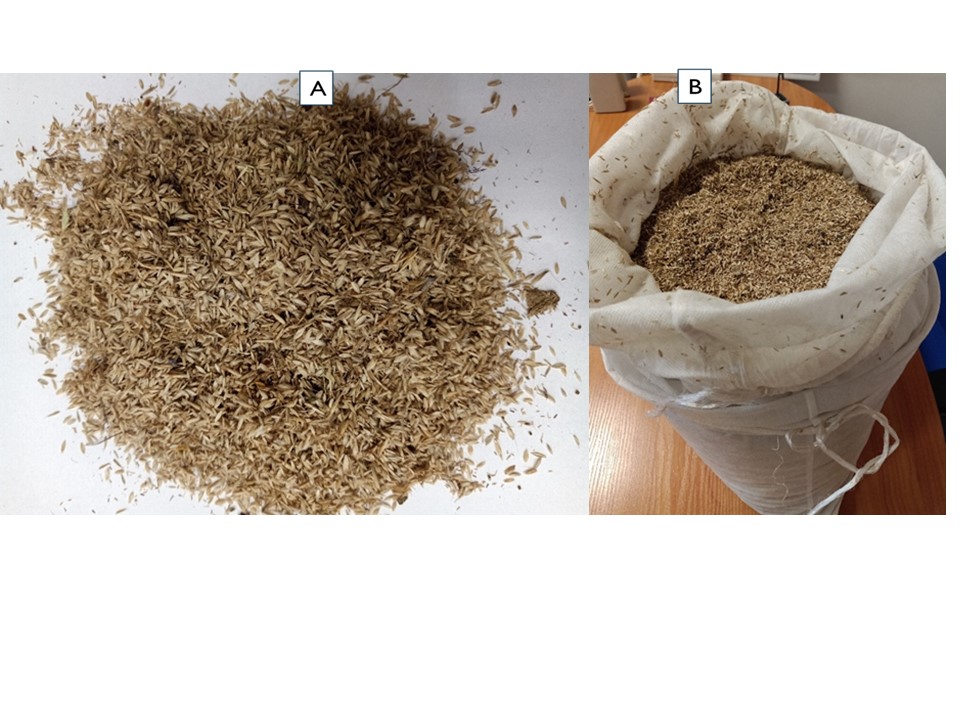

Supplement: Figure S2 [file peerj-10-13621-s003.jpg]

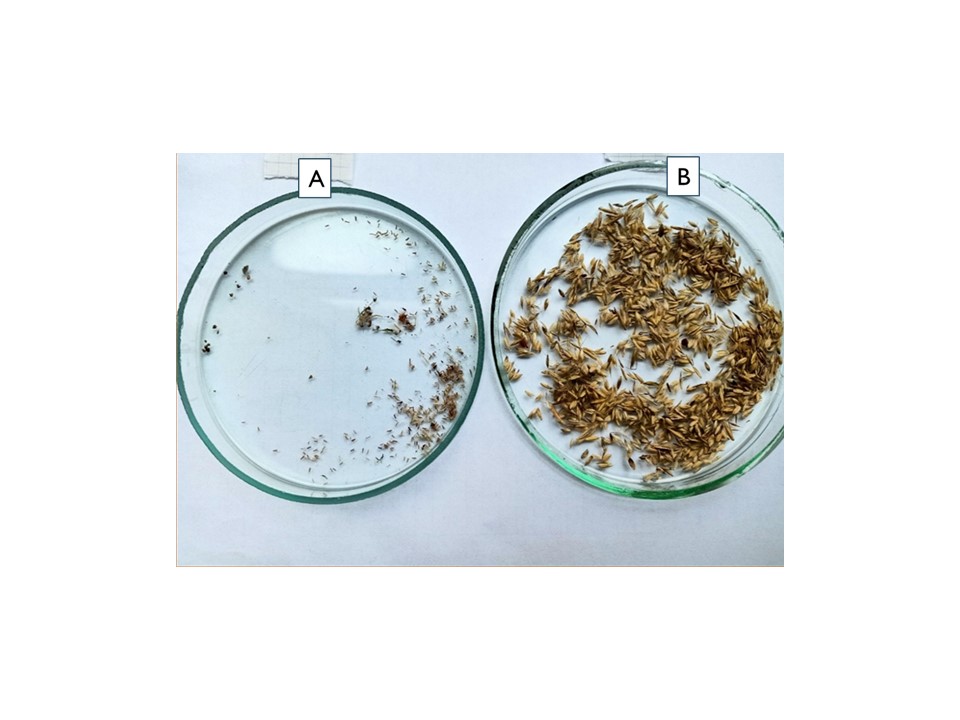

Supplement: Figure S3 [file peerj-10-13621-s004.jpg]

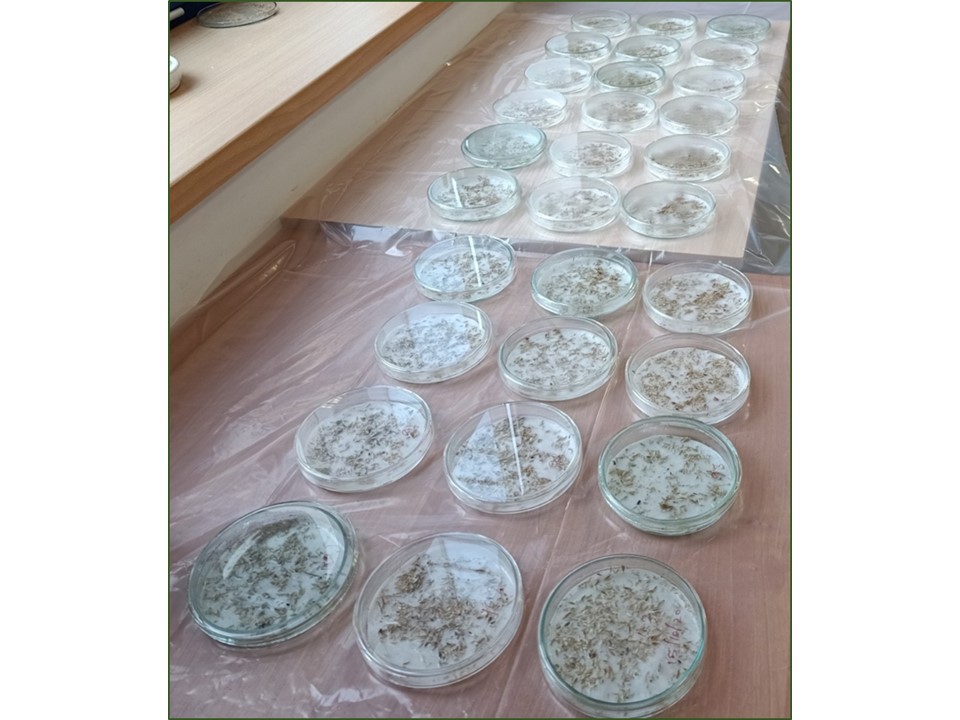

Supplement: Figure S4 [file peerj-10-13621-s006.jpg]

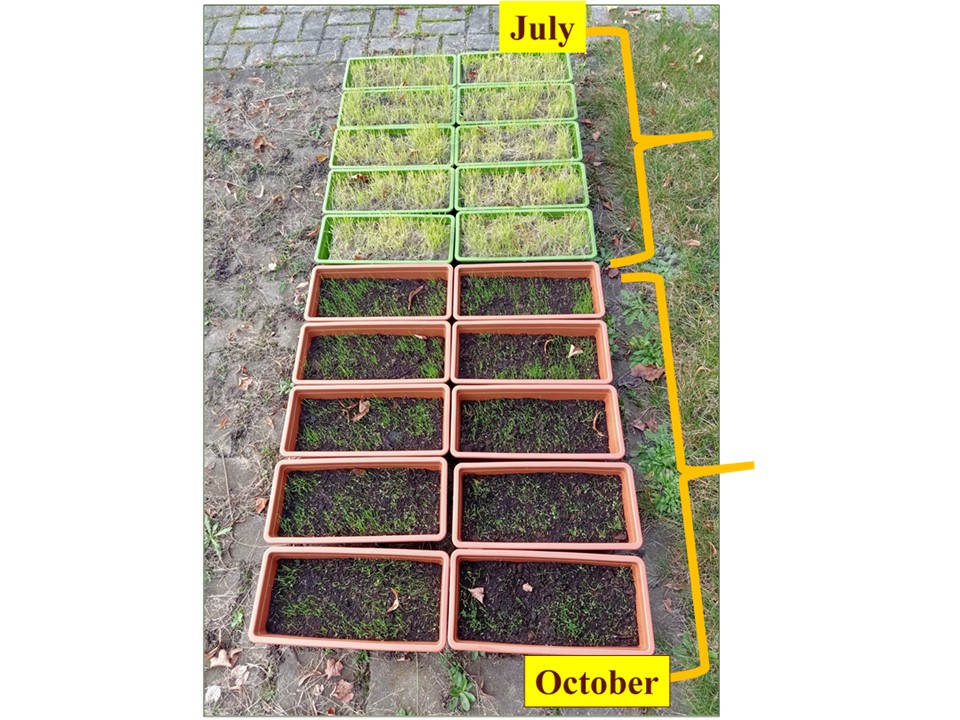

Supplement: Figure S5 — Seeds were sown in two periods in July and October of 2020 (Photos –PCD Perera) [file peerj-10-13621-s007.jpg]

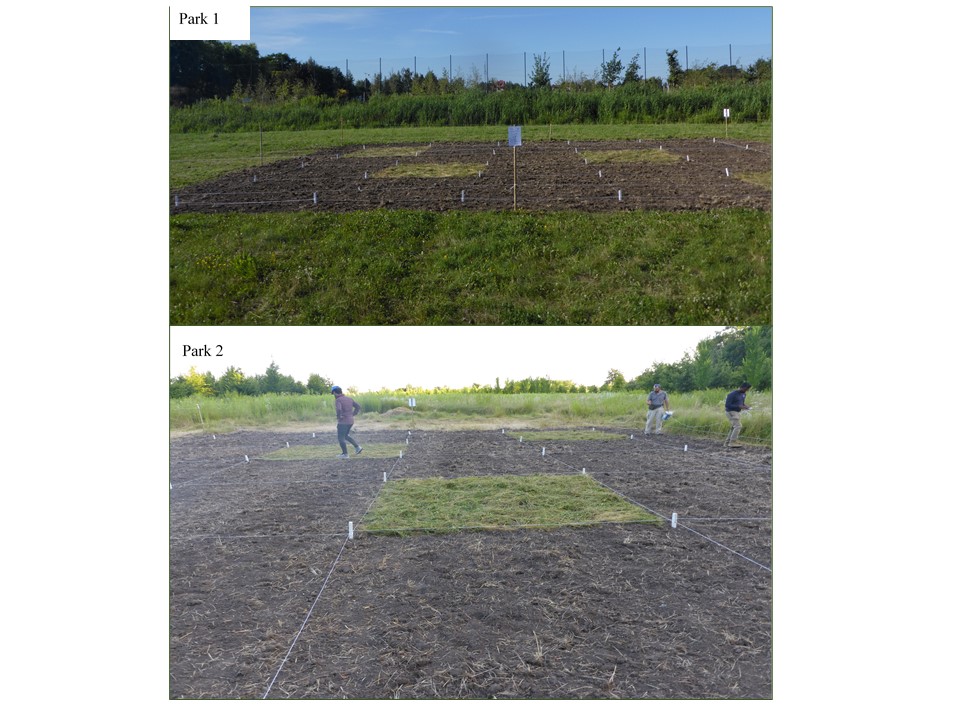

Supplement: Figure S6 — Seeds were seeded in September 2020. These photos are after hay application in July 2020 (Photos –PCD Perera). [file peerj-10-13621-s008.jpg]

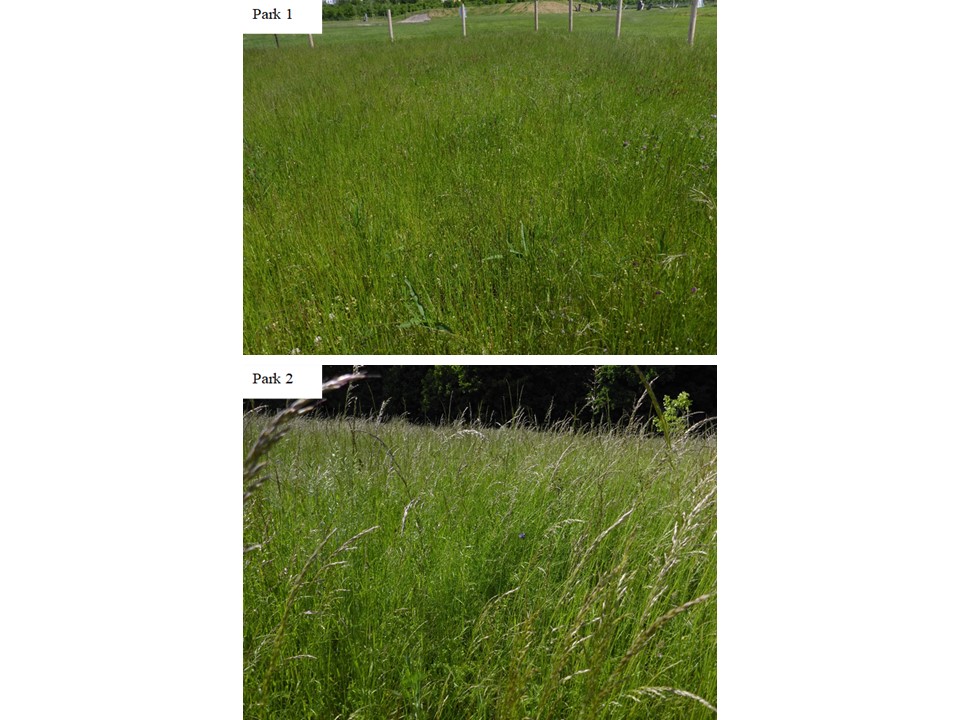

Supplement: Figure S7 [file peerj-10-13621-s009.jpg]
