# Supplementary material for: Seeds harvested during mowing from semi-natural grasslands as an ad hoc but effective solution for grassland restoration
Source: PeerJ. 2022 Jul 13;10:e13621. doi: 10.7717/peerj.13621 (PMC9288168; doi:10.7717/peerj.13621)
Supplement: Table S1 — Species confirmed in the experiments are marked in gray. [file peerj-10-13621-s005.docx]

**Table S1.** Vegetation composition of the donor site (Radomierz) in 2020. Species confirmed in the experiments are marked in gray.

| **Species** | **Frequency** | **Cover** |  | **Species** | **Frequency** | **Cover** |
| --- | --- | --- | --- | --- | --- | --- |
| *Achillea millefolium* L. | 0.9 | 2 |  | *Hypericum maculatum* Crantz | 0.6 | 2 |
| *Aegopodium podagraria* L. | 0.2 | 0.1 |  | *Hypericum perforatum* L. | 0.1 | 0.5 |
| *Agrostis capillaris* L. | 0.9 | 9 |  | *Hypochaeris radicata* L. | 0.1 | 0.1 |
| *Agrostis gigantea* Roth | 0.2 | 3 |  | *Juncus effusus* L. | 0.1 | 0.5 |
| *Alchemilla monticola* Opiz | 0.8 | 2 |  | *Knautia arvensis* (L.) DC. | 0.2 | 0.5 |
| *Alopecurus pratensis* L. | 0.5 | 4 |  | *Lathyrus pratensis* L. | 0.7 | 1 |
| *Angelica sylvestris* L. | 0.5 | 1 |  | *Leontodon autumnalis* L. | 0.1 | 0.1 |
| *Anthoxanthum odoratum* L. | 0.9 | 4 |  | *Leucanthemum vulgare* Lam. | 0.5 | 4 |
| *Anthriscus sylvestris* (L.) Hoffm. | 0.5 | 3 |  | *Lolium perenne* L. | 0.1 | 0.1 |
| *Anthyllis vulneraria* L. | 0.1 | 0.1 |  | *Lotus*  *pedunculatus* Cav. | 0.1 | 0.1 |
| *Arrhenatherum elatius* (L.) J.Presl & C.Presl | 0.8 | 10 |  | *Lotus corniculatus* L. | 0.3 | 0.1 |
| *Artemisia vulgaris L.* | 0.1 | 0.1 |  | *Luzula multiflora* (Ehrh.) Lej. | 0.3 | 0.5 |
| *Brachypodium sylvaticum* (Huds.) P.Beauv. | 0.1 | 0.5 |  | *Lychnis flos-cuculi* L. | 0.2 | 0.1 |
| *Briza media* L. | 0.1 | 0.5 |  | *Myosotis scorpioides* L. | 0.1 | 0.5 |
| *Bromus hordeaceus L.* | 0.1 | 2 |  | *Phleum pratense* L. | 0.5 | 8 |
| *Calystegia sepium* (L.) R.Br. | 0.1 | 0.5 |  | *Pimpinella major* (L.) Huds. | 0.1 | 0.1 |
| *Campanula patula* L. | 0.5 | 0.5 |  | *Pimpinella saxifraga* L. | 0.1 | 0.1 |
| *Campanula trachelium* L. | 0.1 | 0.1 |  | *Plantago lanceolata* L. | 0.9 | 2 |
| *Carex acuta* L. | 0.1 | 3 |  | *Poa palustris* L. | 0.1 | 0.1 |
| *Carlina vulgaris* L. | 0.1 | 2 |  | *Poa pratensis* L. | 0.8 | 4 |
| *Centaurea jacea* L. | 0.4 | 1 |  | *Poa trivialis* L. | 0.2 | 0.1 |
| *Cephalanthera longifolia* (L.) R.M.Fritsch | 0.1 | 0.1 |  | *Polygala vulgaris* L. | 0.1 | 0.1 |
| *Cerastium fontanum* Baumg. | 0.2 | 0.1 |  | *Polygonum persicaria* L. | 0.2 | 0.1 |
| *Cirsium arvense* (L.) Scop. | 0.1 | 0.5 |  | *Ranunculus acris* L. | 0.9 | 1 |
| *Cirsium oleraceum* (L.) Scop. | 0.1 | 0.5 |  | *Ranunculus ficaria* L. | 0.1 | 0.1 |
| *Convolvulus arvensis* L. | 0.1 | 0.5 |  | *Ranunculus repens* L. | 0.5 | 1 |
| *Coronilla varia* L. | 0.1 | 0.1 |  | *Rhinanthus minor* L. | 0.6 | 7 |
| *Crataegus monogyna* Jacq. | 0.1 | 0.1 |  | *Rosa canina* L. | 0.1 | 0.1 |
| *Crepis biennis* L. | 0.5 | 0.5 |  | *Rubus* sp. | 0.2 | 0.5 |
| *Dactylis glomerata* L. | 0.9 | 13 |  | *Rumex acetosa* L. | 0.8 | 2 |
| *Daucus carota* L. | 0.5 | 1 |  | *Rumex obtusifolius L.* | 0.2 | 0.1 |
| *Deschampsia caespitosa* (L.) P. Beauv. | 0.1 | 0.1 |  | *Sanguisorba officinalis* L. | 0.1 | 2 |
| *Dianthus deltoides* L. | 0.1 | 0.5 |  | *Scirpus sylvaticus* L. | 0.1 | 2 |
| *Elymus repens* (L.) Gould | 0.1 | 0.5 |  | *Scrophularia nodosa* L. | 0.2 | 0.1 |
| Equisetum palustre L. | 0.2 | 0.1 |  | *Stellaria holostea* L. | 0.1 | 0.1 |
| *Festuca pratensis* Huds. | 0.9 | 5 |  | *Stellaria media* (L.) Cirillo | 0.8 | 2 |
| *Festuca rubra* L. | 1 | 12 |  | *Tanacetum vulgare* L. | 0.2 | 0.5 |
| *Filipendula ulmaria* (L.) Maxim. | 0.2 | 0.1 |  | *Taraxacum officinale* (L.) F.H.Wigg | 0.5 | 0.5 |
| *Galeopsis tetrahit* L. | 0.2 | 0.1 |  | *Thymus pulegioides* L. | 0.1 | 0.5 |
| *Galium aparine* L. | 0.1 | 0.1 |  | *Tragopogon pratensis* L. | 0.2 | 0.1 |
| *Galium mollugo* L. | 0.5 | 5 |  | *Trifolium pratense* L. | 0.9 | 3 |
| *Galium palustre* L. | 0.1 | 0.5 |  | *Trifolium repens* L. | 0.9 | 1 |
| *Galium uliginosum* L. | 0.1 | 0.1 |  | *Trisetum flavescens* (L.) P.Beauv. | 1 | 15 |
| *Geranium pratense* L. | 0.1 | 0.5 |  | *Veronica chamaedrys* L. | 1 | 2 |
| *Heracleum sphondyliu* L. | 0.4 | 0.1 |  | *Vicia cracca* L. | 0.6 | 0.5 |
| *Hieracium pilosella* L. | 0.1 | 0.5 |  | *Vicia hirsuta* (L.) Gray | 0.5 | 1 |
| *Holcus lanatus* L. | 0.5 | 2 |  | *Vicia sepium* L. | 0.5 | 1 |
| *Holcus mollis* L. | 0.4 | 1 |  |  |  |  |
